# Seroprevalence and risk factors of *Toxoplasma gondii* infection in pregnant women from Bobo Dioulasso, Burkina Faso

**DOI:** 10.1186/s12879-017-2583-6

**Published:** 2017-07-11

**Authors:** Sanata Bamba, Mamoudou Cissé, Ibrahim Sangaré, Adama Zida, Souleymane Ouattara, Robert T. Guiguemdé

**Affiliations:** 1 Université polytechnique de Bobo-Dioulasso, 01 BP 390, Centre MURAZ, Bobo-Dioulasso, Burkina Faso; 2 Université Ouaga 1 Joseph-KI Zerbo, Ouagadougou, Burkina Faso

**Keywords:** *Toxoplasma gondii*, Seroprevalence, Pregnant women, Risk factors, Burkina Faso

## Abstract

**Background:**

Toxoplasmosis is one of the common worldwide parasitic zoonosis due to *Toxoplasma gondii* (*T. gondii)*. Toxoplasmosis during pregnancy can result in fetal and neonatal death or various congenital defects. The aim of this study was to assess the seroprevalence and risk factors of *T. gondii* infection in pregnant women following antenatal care (ANC) services at Bobo Dioulasso.

**Methods:**

A cross-sectional study was conducted enrolling a sample of 316 pregnant women attending ANC at centers for maternal and child health of Bobo-Dioulasso town from March 2013 to February 2014. Data on socio-demographic and potential risk factors were collected from each study participant using structured questionnaire through face-to-face interview. Moreover, venous blood specimens were collected and tested for IgM and IgG anti-*T. gondii* antibodies by enzyme-linked immunosorbent assay and enzyme linked fluorescent assay, respectively. Multivariable logistic regression modeling was used to identify the potential predictor variables for *T. gondii* infection.

**Results:**

The overall seroprevalence for *T. gondii* infection was 31.1% (98/316). All the pregnant women were positive for IgG anti-bodies exclusively. Multivariable logistic regression analysis showed that having at least a secondary education level (AOR = 2.23; 95% CI: [1.04–4.63]); being urban resident (AOR = 2.81; 95% CI: [1.24–6.86]) and the consumption of meat combination (pork + beef + mutton + wild meat + poultry) (AOR = 4.00; 95% CI: [1.06–15.24]) were potential risk factors of *T. gondii* infection.

**Conclusion:**

Toxoplasmosis is frequent in pregnant women and studies that show incidence of *T. gondii* among the neonates have to be done to introduce routine antenatal screening program to control congenital toxoplasmosis. There is the need for preventive measures such as education of pregnant women about the transmission routes and prevention methods of toxoplasmosis at ANC clinics.

## Background

Toxoplasmosis is one of the common worldwide parasitic zoonosis, caused by an obligate intracellular protozoan parasite *Toxoplasma gondii* [1]. Felines are the only definitive host while all other warm-blooded animals including humans are intermediate hosts for the parasite [2]. *T. gondii* is commonly transmitted to humans by accidental ingestion of oocyst stage of the parasite in water, food or soil contaminated with cat’s faeces, or by eating raw or undercooked meat containing oocysts [2, 3]. It can also be transmitted congenitally during pregnancy [4]. In addition, other infectious pathways include blood transfusion, and organs transplantation [5]. Toxoplasmosis is more common in areas with tropical and very humid climates which are favorable conditions for the maintenance and dissemination of the oocysts [6].

Toxoplasmosis is a major public health problem in the world. Indeed, it is estimated that about one third of the World’s population is infected with *T. gondii.* Although usually asymptomatic, it can result during pregnancy in fetal and neonatal death or various congenital defects [7] especially when the congenital infection occurs during the first trimester due to acute infection during pregnancy.

In Africa, the seroprevalence of *T. gondii* during pregnancy is generally as high as 80% [3, 8]. Moreover, presence of domestic cat at home [3], contact with cat and gardening soil [8] were found to be the main risk factor toxoplasmosis during pregnancy.

In Burkina Faso, the seroprevalence of toxoplasmosis during pregnancy have been poorly reported [9–11] and none of those previous reports had assessed risk factors for toxoplasmosis. This study sought therefore to determine the seroprevalence of *T. gondii* and to identify the potential risks factors associated with of *T. gondii* infection among pregnant women following ANC services at Bobo Dioulasso, the second largest city of Burkina Faso.

## Methods

### Study area and period

This study was conducted in Bobo-Dioulasso town from March 2013 to February 2014. Bobo-Dioulasso is the second biggest city of Burkina Faso located in the South-west of the country with an estimated population of 1.7 million inhabitants. The climate is subtropical and humid with an average annual temperature above 20 °C. Agriculture (e.g., corn, millet, sorghum, peanuts, rice, cotton, vegetables) and livestock (poultry and cattle) is the main economic activity. Also, water supply system is made of natural source and drilling. Once collected, water will undergo several types of treatment depending on their origin so that it can be suitable for consumption and is then distributed to the population according to standards set by the World Health Organization (WHO). Overall 5000 pregnant women attended the ANC clinics per year with an estimated birth rate of 43.6 ‰ [12]. There was no serological screening of pregnant women for *T. gondii* infection in Bobo-Dioulasso town and Burkina Faso in general.

### Study design and population

We carried out a cross-sectional study enrolling a sample of 316 pregnant women attending ANC at centers for maternal and child health of Bobo-Dioulasso town from March 2013 to February 2014. Sample size was determined using single population proportion formula with seroprevalence value (*p* = 30.0%) taken from previous study (10) and 95% confidence interval (CI) with a 5% desired absolute precision was considered. A systematic random sampling technique was used to obtain the required sample.

### Questionnaire survey

Pregnant women were interviewed to collect data about the potential risk factors for toxoplasmosis by using structured questionnaire. The questionnaire covered socio-demographic information including age, level of education, occupation, residence (urban area versus rural area of Bobo-Dioulasso town (i.e. those who are living in the suburb of the city)), source of drinking water, and obstetric history and other behavioral factors like consumption of meat and milk. Furthermore, presence of domestic cats in their home was addressed.

### Samples collection and serological analysis

After signing informed consent, 10 mL of venous blood were collected from each pregnant women in the tube coated EDTA. Each tube was centrifuged at 3000 rpm for 10 min to remove plasma that was frozen at −20 °C until use.

Serogical diagnosis of toxoplasmosis was performed using both immunoglobin G (IgG) and immunoglobulin M (IgM) antibodies determination from sera in the Laboratory of Parasitology and Mycology of the Sourou Sanou teaching hospital of Bobo-Dioulasso. Sera were analysed using a standard Enzyme-linked immunosorbent assay (ELISA) commercial Kit (AxSYM), Abbott, Laboratoiries, Abbott Park, III, USA) and according to the manufacturer’s instructions. A titer of IgG *anti-Toxoplasma* antibody ≥3 IU/ml was considered as positive in this study [13]. In order to confirm the first results, new samples were collected after 21 days.

Quantitative determination of specific IgM antibodies was performed using the enzyme linked fluorescent assay (ELFA) based on the VIDAS System (bio Mérieux-Lyon, France). IgM concentration > 0.65 index was used as reference value for positive results. Samples were considered reactive for IgM when the antibody concentration was greater than or equal to 0.6 index, non-reactive when the concentration was equal to 0 index, and undetermined when the concentration was between 0.4 and 0.5 index.

IgG avidity index was measured using the VIDAS System (bio Mérieux-Lyon, France) and results were interpreted according to the manufacturer’s instructions with the reference value for high avidity i.e., IgG > 0.3 [14].

### Data analysis

Data were coded and entered into Epidata 3.1 software. Data were thereafter imported to statistical STATA 12 (Stata Corp, College Station, TX, USA), cleaned before performing analyses. Cross-tabulations of sero-status were done with socio-demographic and behavioral characteristic as summary measures.

Univariate logistic regression was used as bivariate analysis to select significant variables to be used in subsequent multivariable logistic regression analysis. Multivariable logistic regression analysis was used to calculate adjusted odds ratios (AOR) and computed their 95% CI. Variables resulting in *p*-values less than 0.05 was considered to be significantly associated with seroprevalence of toxoplasmosis.

### Ethical considerations

Ethical clearance was obtained from the National Research Ethical Committee from Burkina Faso (number 0123/10). Informed written consent was sought from each pregnant woman prior to involvement in the study. Information collected from each study participant was kept confidential and venous blood specimens collected were preserved anonymously.

## Results

### Seroprevalence of *T. gondii* infection

A total of 316 pregnant women were enrolled during the study period with the mean age ± standard deviation (SD) of 26. 9 ± 4.2 years. The prevalence of specific anti-*T. gondii* IgG was of 31.1% (98/316). No IgM antibodies for *T. gondii* were found. All cases with *Toxoplasma*-specific antibodies presented high avidity indexes.

### Risk factors associated with *T. gondii* infection

In univariate analysis only non-housewives (OR = 2.05; 95% CI: [1.18–3.57]), women with at least secondary education level (OR = 2.12; 95% CI: [1.07–4.18]) and those living in urban areas of Bobo-Dioulasso town (OR = 2.41; 95% CI: [1.07–5.42]) were at higher odds of *T. gondii* infection (Table 1). In addition, consumption of meat combination (pork + beef + mutton + bush meat + poultry) was significantly associated with *T. gondii* seroprevalence (OR = 4.78; 95% CI: [1.31–17.30]) (Table 2).

**Table 1:** Univariate analysis of *Toxoplasma* seroprevalence in relation to socio-demographic characteristics among the pregnant women (*n* = 316) in Bobo-Dioulasso town

Variable	*Toxoplasma* seroprevalence	Crude OR, CI 95%	*P-value*
Age category Age			
17–19	32.7 (18/55)	1	-
20–29	32 (68/212)	0.96 [0.40–1.78]	0.87
> 30	35.3 (12/34)	0.97 [0.39–2.44]	0.97
Occupation			
Housewife	27.8 (64/230)	1	-
Others^a^	48.6 (34/70)	2.05 [1.18–3.57]	0.01
Education level			
Illiterate	28.2 (49/174)	1	-
Primary	35.4 (29/82)	1.17 [0.66–2.10]	0.56
At least secondary	45.5 (20/44)	2.12 [1.07–4.18]	0.03
Marital statut			
Married	32.3 (81/251)	1	-
Unmarried	36.1 (17/47)	0.71 [0.34–1.47]	0.36
Residence			
Rural areas	30.6 (27/88)	1	-
Urban residence	33.5 (71/212)	2.41 [1.07–5.42]	0.03
Number of gravida			
One	22.2 (10/45)	1-	-
More than one	34.4 (88/256)	1.34 [0.77–2.23]	0.29
Water supply			
General distribution	32.3 (94/291)	1	-
Bottled water	3.33 (1/3)	1.10 [0.10–11.26]	0.94
Other source^b^	60.0 (3/5)	3.31 [0.53–20.06]	0.20
Presence of cat at home			
No	29.6 (75/253)	1	-
Yes	48.9 (23/47)	1.75 [0.92–3.32]	0.08

^a^students, employees and unemployed; ^b^Well or pond

**Table 2:** Univariate analysis of *Toxoplasma* seroprevalence in relation to behavioral characteristics among the pregnant women (*n* = 316) in Bobo-Dioulasso town

Variable	*Toxoplasma* seroprevalence	Crude OR, 95% CI	*P-value*
Type of meat consumed			
Beef	21.2 (7/33)	1	-
Mutton	16.7 (3/18)	0.73 [0.17–3.31]	0.70
Poultry	34.2 (75/219)	1.82 [0.75–4.38]	0.18
Pork	28.6 (4/14)	1.49 [0.35–6.20]	0.59
Meat combination^a^	56.3 (9/16)	4.78 [1.31–17.30]	0.02
Frequency of meat consumption			
Regular	31.3 (63/201)	1	-
Irregular	35.0 (35/100)	1.07 [0.65–1.70]	0.77
Degree of meat cooking			
Well-cooked	31.6 (6/19)	1	-
Undercooked	32.6 (92/282)	1.01 [0.37–2.75]	0.98
Consumption of milk			
No	32.8 (20/61)	1	-
Yes	32.5 (78/240)	0.96 [0.51–1.72]	0.87

^a^pork + beef + mutton + bush meat + poultry

Finally, the result of multivariable logistic regression analysis showed that having at least secondary education level (AOR = 2.23; 95% CI: [1.04–4.63]); urban residence (AOR = 2.81; 95% CI: [1.24–6.86]) and the consumption of meat combination (pork + beef + mutton + wild meat + poultry) (AOR = 4.00; 95% CI: [1.06–15.24]) were potential risk factors of *T. gondii* infection (Table 3).

**Table 3:** Risk factors associated with *Toxoplasma* seroprevalence among the pregnant women (*n* = 316) in Bobo-Dioulasso town

Variable	*Toxoplasma* seroprevalence	Adjusted OR 95% CI	*P-value*
Occupation			
Housewife	27.8 (64/230)	1	-
Others	48.6 (34/70)	1.83 [1.0–3.40]	0.05
Education level			
Illiterate	29.8 (52/174)	1	-
Primary	31.7 (26/82)	1.10 [0.61–2.00]	0.75
At least secondary	45.5 (20/44)	2.23 [1.04–4.63]	0.04
Residence			
Rural areas	27.3 (24/88)	1	-
Urban residence	34.9 (74/212)	2.81 [1.24–6.86]	0.01
Presence of cat at home			
No	29.6 (75/253)	1	-
Yes	48.9 (23/47)	1.61 [0.81–3.18]	0.16
Type of meat consumed			
Beef	21.2 (7/33	1	-
Mutton	33.3 (6/18)	0.51 [0.11–2.38]	0.42
Poultry	32.9 (72/219)	1.66 [0.67–4.11]	0.27
Pork	28.6 (4/14)	1.04 [0.24–4.48]	0.96
Meat combination^a^	56.3 (9/16)	4.00 [1.06–15.24]	0.04

^a^pork + beef + mutton + bush meat + poultry

## Discussion

The prevalence of anti-*T. gondii* IgG antibodies observed in this study (31.1%) was consistent with previous findings reported in pregnant women from urban areas of Burkina Faso and ranged from 28.3% to 31% [9–11]. Our result was lower than those reported in Ghana (92.5%) [15] and in Ethiopia (higher than 68%) [3, 8, 16, 17]. However, lower seroprevalence of *T. gondii* was reported in many European countries and the United States of America [18]. Such seroprevalence variations could be due to differences in climatic conditions (dry climate, rainfall, temperature, soil type, altitude) and mothers’ characteristics such as management of cats, educational level, hygienic practice and feeding habit [3, 8, 17]. Furthermore, differences in serological methods used and the sensitivity difference could explain such discrepancy [8, 17].

All the pregnant women were positive for IgG anti-bodies exclusively suggesting either past infection or acquired immunity. Thus, they were not likely able to infect their fetus outside a context of high immunosuppression [6, 19].

In this study high education level, being urban resident and the consumption of meat combination (pork + beef + mutton + wild meat + poultry) were identified as possible risk factors associated with *T. gondii* infection. Women with high education level were more likely to consume meat combination (pork + beef + mutton + wild meat + poultry) probably due to their high income and were therefore more exposed to *T. gondii* infection. Our findings suggest the need for preventive measures such as education of pregnant women about the transmission routes and prevention methods of toxoplasmosis at ANC clinics. However, a previous study in Ethiopia showed that women who cannot read and write were 5 times more likely to acquire *T. gondii* infection than those who had tertiary level of education [17]. In Brazil, pregnant women with low educational status had higher seroprevalence of *T. gondii* [20]. Moreover, reports from Netherlands and USA, showed that the seroprevalence of toxoplasmosis increases when the level of education decreases [6, 21].

Furthermore, the association between seroprevalence of *T. gondii* and urban residence of Bobo-Dioulasso town could be explained by insalubrity due to the demographic pressure in that town and dilapidation of water pipelines. Both factors are known to favor the survival of oocysts and to maintain a high prevalence of *T. gondii* infection [22–24]. Our results suggest the need for conducting further studies to better understand to what extend urban residence plays a role in the epidemiology of toxoplasmosis in Bobo-Dioulasso town.

In our study pregnant women consuming meat combination (pork + beef + mutton + wild meat + poultry) were significantly at greater risk of toxoplasmosis. This could be due to differences in the seroprevalence of *T. gondii* in the type of animals consumed in that town. Indeed, we previously reported a seroprevalence of 14.3% and 60% for both bovine and ovine toxoplasmosis in Bobo-Dioulasso town during the same period of the study [25, 26]. Altogether our data highlight the need for further studies aiming to assess the seroprevalence of *T. gondii* in domestic animals such pigs and poultry.

We do acknowledge some limitations to our study. First, risk factor assessment was done based on study participants’ response which may suffer from recall bias. Second, since this study was heath centers based study, it may not represent the general population.

## Conclusion

Toxoplasmosis is frequent in pregnant women and studies that show incidence of congenital infection among the neonates have to be done to introduce routine antenatal screening program to control congenital toxoplasmosis. Education level, being urban resident and the consumption of meat combination were identified as possible risk factors associated with *T. gondii* infection. There is the need for preventive measures such as education of pregnant women about the transmission routes and prevention methods of toxoplasmosis at ANC clinics. Further studies aiming to assess the seroprevalence of *T. gondii* in domestic animals are warranted.

## Abbreviation

ANC: Antenatal care
AOR: Adjusted odds ratio
CI: Confident interval
ELFA: Enzyme linked fluorescent assay
ELISA: Enzyme linked immunosorbent assay
IgG: Immunoglobulin G
IgM: Immunoglobulin M
WHO: World Health Organization.
